# Supplementary material for: Effectiveness of Cervical Testing in and outside a Screening Program—A Case-Control Study
Source: Cancers (Basel). 2022 Oct 23;14(21):5193. doi: 10.3390/cancers14215193 (PMC9653595; doi:10.3390/cancers14215193)
Supplement: Supplementary file 1 [file cancers-14-05193-s001.zip › cancers-1922090-supplementary.pdf]

Supplementary Information

Table S1. Sensitivity analysis with complete and restricted follow-up times. Association between testing in a five and three-year interval and cervical cancer diagnosis in the following five-year interval. Adjusted for education, socioeconomic status, and mother tongue.

			5-Year Interval			3-Year Interval	
		Cases, N (%)	Controls, N (%)	OR (95%CI)	Cases, N (%)	Controls, N (%)	OR (95%CI)
End of FU 2019	All ages	1677 (100)	16738 (100)		1677 (100)	16738 (100)	
(full data)	No tests	779 (46)	4920 (29)		854 (51)	6063 (36)	
	Any test	898 (54)	11818 (71)	0.43 (0.38-0.48)	823 (49)	10675 (64)	0.49 (0.44-0.55)
	Screening target ages *	1081 (100)	10793 (100)		1082 (100)	10802 (100)	
	No tests	412 (38)	1807 (17)		435 (40)	2178 (20)	
	Only program	290 (27)	3864 (36)	0.35 (0.29-0.41)	362 (33)	5248 (49)	0.36 (0.31-0.43)
	Only outside	181 (17)	2059 (19)	0.42 (0.35-0.51)	146 (13)	1685 (16)	0.48 (0.39-0.59)
	Both	198 (18)	3063 (28)	0.31 (0.25-0.37)	139 (13)	1691 (16)	0.46 (0.37-0.56)
End of FU 2015	All ages	974 (100)	9716 (100)		974 (100)	9716 (100)	
(restricted) **	No tests	470 (48)	2823 (29)		504 (52)	3501 (36)	
	Any test	504 (52)	6893 (71)	0.37 (0.32-0.44)	470 (48)	6215 (64)	0.46 (0.39-0.54)
	Screening target ages*	605 (100)	6040 (100)		602 (100)	6009 (100)	
	No tests	235 (39)	973 (16)		246 (41)	1180 (20)	
	Only program	143 (24)	1893 (31)	0.32 (0.25-0.41)	179 (30)	2759 (46)	0.31 (0.25-0.39)
	Only outside	98 (16)	1233 (20)	0.35 (0.27-0.46)	83 (14)	1001 (17)	0.43 (0.32-0.56)
	Both	129 (21)	1941 (32)	0.29 (0.23-0.37)	94 (16)	1069 (18)	0.45 (0.34-0.58)

* The national screening target ages at the time of study were 30, 35, …, 55, 60.

** Data on tests outside the program were extensively available until 2014 and to a limited degree until 2016/2017. Sensitivity analysis with comprehensive test data in 2000-2014 enabled cancer surveillance until 2015.

Table S2. Association between testing and cervical cancer diagnosis 5.5 and 3.5 years prior to cases' diagnosis. Adjusted for education, socioeconomic status, and mother tongue. Similar analysis as in article Table 2, but with different definition for the test exposure.

	5.5 Years Prior to Diagnosis			3.5 Years Prior to Diagnosis		
	Cases, N (%)	Controls, N (%)	OR (95%CI)	Cases, N (%)	Controls, N (%)	OR (95%CI)
All ages	1677 (100)	16738 (100)		897 (100)	8999 (100)	
No tests	843 (50)	5876 (35)		591 (66)	4758 (53)	
Any test	834 (50)	10862 (65)	0.48 (0.43-0.54)	306 (34)	4241 (47)	0.49 (0.41-0.59)
Screening target ages*	1076 (100)	10764 (100)		436 (100)	4448 (100)	
No tests	433 (40)	2262 (21)		207 (47)	1175 (26)	
Only program	270 (25)	3728 (35)	0.38 (0.32-0.46)	133 (31)	2142 (48)	0.34 (0.27-0.44)
Only outside	182 (17)	2223 (21)	0.45 (0.37-0.55)	41 (9)	647 (15)	0.39 (0.27-0.56)
Both	191 (18)	2551 (24)	0.41 (0.34-0.5)	55 (13)	484 (11)	0.7 (0.5-0.99)

* The national screening target ages (in the whole country) at the time of study were 30, 35, …, 55, 60.

Note: In this analysis, the time from diagnosis/index date to test was limited to 5.5 or 3.5 years, unlike in the main analysis of the article, where the intervals for tests and cancer diagnoses did not overlap. Among the screening target aged women (30—64-year-olds), data were restricted so that a woman could have had taken both tests during the 5.5/3.5-year interval. Since all tests 12 months prior to the index date were excluded, the youngest index age was 31 so that the earliest test considered for the analysis would be at age 30. For the 5.5-year analysis, the oldest index age was 65.5 and for the 3.5-year analysis, the oldest index age was 63.5, incorporating tests from the last nationwide screening round at age 60. Additional exclusions were made for the 3.5-year analyses, also outside the screening target ages: To make the two testing intervals of interest more comparable, only those eligible to receive a screening invitation within the 3.5-year period were included. That is, index ages 26—28.5, 31—33.5, …, 61—63.5 and 66—68.5 were included. For example, to include the program screen at age 30, diagnosis ages 31—33.5 need to be included but ages 33.5—35 need to be excluded. No exclusions were done at ages <25 or >70.
